# Comparison of new and recurring post-acute infection syndrome – results from a German population-based cohort

**DOI:** 10.1038/s41598-026-70451-3

**Published:** 2026-09-12

**Authors:** Jonas Frost, Friederike Peter, Nadine Glaser, Laura R. Pfrommer, Jonathan Mathias Fasshauer, Nils Opel, Michael Gekle, Thomas Behrens, Oliver Tüscher, Rafael Mikolajczyk

**Affiliations:** 1 Institute for Medical Epidemiology, Biometrics, and Informatics, Interdisciplinary Centre for Health Sciences, Medical Faculty of the Martin Luther University Halle-Wittenberg, Magdeburger Str. 8, 06112 Halle (Saale), Germany; 2 Department of Psychiatry and Neuroscience, German Center for Mental Health (DZPG), Charité University Medical Center, Campus Benjamin Franklin, Hindenburgdamm 30, 12203 Berlin, Germany; 3 Julius-Bernstein-Institute of Physiology, Interdisciplinary Centre for Health Sciences, Medical Faculty of the Martin Luther University Halle-Wittenberg, Magdeburger Str. 6, 06112 Halle (Saale), Germany; 4 Institute for Prevention and Occupational Medicine of the German Social Accident Insurance, Institute of the Ruhr University, Medical Faculty, Bürkle de la Camp-Platz 1, 44789 Bochum, Germany; 5 Department of Psychiatry, Psychotherapy and Psychosomatic Medicine, University Medicine Halle (Saale) of the Martin Luther University Halle-Wittenberg, Julius-Kühn-Str. 7, 06112 Halle (Saale), Germany; 6 German Center for Mental Health (DZPG), Partner site Halle-Jena-Magdeburg, Halle (Saale), Germany

**Keywords:** Post-acute infection syndrome, Reinfection, Respiratory infection, Diseases, Health care, Medical research, Signs and symptoms

## Abstract

New respiratory infections after or while experiencing post-acute infectious syndromes (PAIS) can lead to resurgence of symptoms. Data comparing symptoms, related impairment, and duration of first and recurring PAIS episodes are lacking. This cross-sectional study was conducted in September/October 2025 within a large population-based digital cohort in Germany (DigiHero, registered under DRKS00025600). We included data from 2,801 participants retrospectively reporting a respiratory infection between September 2024 and August 2025 with subsequent long-lasting symptoms ≥ 12 weeks after infection. We compared self-reported first and recurring PAIS episodes. Of detected PAIS cases, 57% (*n* = 1,609) were the first episode and 43% (*n* = 1,192) already experienced a PAIS episode in the past. While symptom patterns were broadly similar, those reporting recurring PAIS without full recovery from a previous episode (*n* = 308) displayed a higher number and longer duration of long-lasting symptoms and a higher level of impairment due to their symptoms compared to those with first PAIS episodes or recurring cases that were fully recovered at the time of the new infection (*n* = 303). Among participants with recurring PAIS who compared their most recent with their first episode, 43% reported similar impairment, while 32% reported greater and 26% lower impairment. While after the COVID-19 pandemic PAIS is still occurring, a substantial proportion of all PAIS cases in 2024/25 in the DigiHero cohort resulted from those with recurring PAIS. Symptom profiles were in general similar for first and recurring PAIS episodes. However, recurring cases exhibited greater impairment and longer symptom duration, when they did not fully recover from a preceding PAIS episode before the new infection.

**Supplementary Information:**

The online version contains supplementary material available at https://doi.org/10.1038/s41598-026-70451-3.

## Introduction

Post-acute infection syndromes (PAIS) are characterised by persistent or new symptoms following an acute infection. During the SARS-CoV-2 pandemic, increased attention was directed toward PAIS in the context of COVID-19, commonly referred to as long-COVID or post-COVID syndrome. Due to the large number of SARS-CoV-2 infections worldwide, post-COVID has contributed to a measurable burden on healthcare systems and societies^1–3^.

In the post-pandemic phase, SARS-CoV-2 has become one of several circulating respiratory pathogens, alongside influenza viruses and respiratory syncytial virus (RSV)^4^. As a result, interest has increasingly shifted from post-COVID alone towards PAIS as a broader sequela of infectious diseases. This perspective aligns with earlier work on post-infectious syndromes following other viral and non-viral infections^5–8^. Hickie et al. (2006) already observed persisting post-infective fatigue and prolonged illness following infections with several different pathogens including Epstein Barr virus, Ross River virus and Q fever^5^, while Miller et al. (2026) reported that PAIS was described as early as 1890 during the Russian influenza epidemic^6^. More recent studies highlight the shared core of common symptoms across PAIS triggered by various causative agents^7,8^.

While for post-COVID, several clinical and research definitions have been proposed describing it as symptoms occurring within three months of probable or confirmed SARS-CoV-2 infection, lasting for at least two months, and not explained by an alternative diagnosis^9^ and recognising that symptoms may be continuous, relapsing and remitting, or progressive^10^, there is currently no broadly accepted, standardised case definition for PAIS following other infections. In the present study, PAIS is therefore used as an umbrella term for self-reported long-lasting symptoms attributed to a preceding respiratory infection, fulfilling the post-COVID criteria irrespective of the pathogen.

The health-care burden and individual impact of post-COVID, and more recently PAIS in general, have been studied extensively. Most prior studies have focused on risk factors, symptom profiles, duration, and severity of first PAIS episodes following an index infection^11–13^. These studies consistently report substantial heterogeneity in symptom presentation and disease course, including persistent and fluctuating symptom trajectories.

By contrast, less is known about recurring PAIS episodes in individuals with a history of ongoing or previously recovered PAIS. While several studies indicate that reinfection with SARS-CoV-2 is associated with an increased cumulative burden of post-acute sequelae^14–17^, most analyses do not explicitly distinguish between first and subsequent PAIS episodes at the individual level. This distinction is inherently challenging, as current definitions of post-COVID acknowledge that symptoms may fluctuate, relapse, or occur intermittently, making it difficult to differentiate sustained recovery from temporary symptom resolution on the one side and new episode of PAIS or worsening of PAIS after a new infection from just fluctuation in symptoms of PAIS. In general, evidence comparing the severity, duration, and symptom patterns of recurring PAIS episodes with first episodes is lacking.

This analysis addresses this gap by assessing previous recovery and recurrence of PAIS based on participants’ self-reported perception and attribution and comparing severity, duration, and symptom patterns of self-reported recurring PAIS episodes with first PAIS episodes in the population-based German digital cohort study DigiHero, conducted in a post-pandemic setting.

## Methods

### Study design

This study was conducted inside the population-based German digital cohort DigiHero (DRKS Registration-ID: DRKS00025600)^12,16,18^. Recruitment for DigiHero started in 2021 in Saxony-Anhalt and has since then been expanded to all 16 German federal states. Individuals aged 18 and older were randomly sampled from registration offices’ registries and invited to participate via postal mail, resulting in a population-based design. Additionally, individuals can self-register on the study’s website. After registration, participants filled in an online questionnaire assessing sociodemographic variables like sex, age and federal state of residence. Subsequently, participants are invited to online surveys on health-related topics multiple times per year. The study was approved by the Ethics Committee of the Martin Luther University Halle-Wittenberg, Germany (No. 2020-076) and performed in accordance to all relevant ethical and data protection regulations. Informed consent was obtained online from all participants.

In September 2025, we conducted a short cross-sectional survey of all 128,748 registered participants on respiratory infections between September 2024 and August 2025 and if they experienced any long-lasting symptoms ≥ 12 weeks after an infection in that time period. Participants reporting an infection and subsequent long-lasting symptoms were then invited to a second survey (translated English questionnaires available in Supplementary Material S1, timeline of assessments in supplementary Figure S1). This cross-sectional follow-up questionnaire first verified if participants self-attributed their long-lasting symptoms to the infection. In the follow-up, participants were asked if they had experienced an episode of long-lasting symptoms after an infection before September 2024 already (binary assessment). After about half the participants were invited to the follow-up questionnaire, we added questions on the number of prior PAIS episodes, if participants had fully recovered from the last episode at the time of the new infection (binary assessment) and how they compare the severity of current symptoms to their first episode of PAIS (5-point scale ranging from “much less severe” to “much more severe”). This allowed us to differentiate four groups of participants with an infection and subsequent long-lasting symptoms in the study period: (1) individuals that experienced their first PAIS episode, (2) individuals that experienced a recurrence of PAIS but had fully recovered from their last episode, (3) individuals with recurring PAIS whose symptoms from the last episode had not fully ceased, and (4) participants with recurring PAIS for whom the prior status of recovery was not assessed.

Further, participants were asked about the date of the initial infection and the severity of the acute infection (5-point scale from “very mild” to “very severe”), the number of prior SARS-CoV-2 infections (as recalled) and vaccinations, whether they received a vaccination against influenza in the 2024/2025 season (binary assessment) and if any test was performed either self-administered or by a physician. If they reported a test, we also asked to self-report its result (influenza, RSV, SARS-CoV-2, other pathogen, or unknown). To determine the most likely pathogen among those reporting a test result, tests performed by a physician were the primary source of information and only if no result was reported, results from self-tests were used. The follow-up questionnaire further assessed what symptoms were present ≥ 12 weeks after infection (list of 24 symptoms available in Supplementary Materials S1, assessed in three categories “not present”, “present but not related to the infection”, “present and related to the infection”). For analyses of PAIS symptoms, only symptoms reported as present and related to the infection were coded as present. Using these 24 symptom variables, we calculated the number of reported symptoms. Additionally, it included a question on the impairment experienced due to these long-lasting symptoms (6-point scale ranging from “not at all” to “very strongly” impaired). Also, participants were asked if the symptoms were still ongoing or when they ceased. Results on the incidence of PAIS by age as well as characteristics of PAIS cases by pathogen using the same data were reported in a previous manuscript^19^.

In the current study, analyses were conducted for all participants reporting at least one respiratory infection with subsequent long-lasting symptoms ≥ 12 weeks after infection in the first survey, whose infection was more than 12 weeks before the questionnaire and who further confirmed a self-perceived association of long-lasting symptoms with the infection in the follow-up. Participants with missing age and sex were excluded from the study.

### Statistical analyses

First, the study population was described with respect to sex and age, the reported pathogen of the recent respiratory infection, severity of the acute infection, influenza vaccination in the last winter season (absolute counts and percentages), number of prior SARS-CoV-2 infections and vaccinations (mean and standard deviation (SD)). Then, participants that experienced their first PAIS episode (first PAIS) and participants that experienced a recurrence of PAIS (recurring PAIS after recovery, not recovered and unknown) were compared regarding their reported impairment due to the long-lasting symptoms, the number of reported symptoms and symptom patterns as well as the duration of symptoms. For binary variables, proportions and 95% confidence intervals (95%-CIs) were computed using the exact binomial (Clopper–Pearson) method. For the number of symptoms, group means and corresponding 95%-CIs based on Student’s *t* distribution were reported. The duration of symptoms was compared using Kaplan-Meier curves and corresponding 95%-CIs.

Additionally, to verify observed differences in these outcomes (impairment, number of symptoms, symptom duration) adjusted for group differences, we performed multivariable regression analyses. All three regression models were adjusted for age (modelled continuously per ten-year increase), sex, self-reported number of previous SARS-CoV-2 vaccinations (lifetime), influenza vaccination in the last winter season, number of prior SARS-CoV-2 infections (self-reported, lifetime), the reported pathogen of the recent infection and the severity of acute symptoms. For impairment, a multivariable ordinal logistic regression was performed. For the number of symptoms, a multivariable linear regression was performed and for symptom duration, a Cox proportional hazard regression model was computed. Single symptom prevalences were adjusted using symptom-wise logistic regressions keeping the covariates constant across groups and symptoms. Predicted prevalences for the four groups were evaluated descriptively by comparing the adjusted prevalence estimates and their 95%-CIs.

Model assumptions and stability were assessed using tests of the proportional-odds and proportional-hazards assumptions, graphical residual diagnostics, tests for heteroscedasticity, and assessment of multicollinearity. Partial proportional-odds and zero-truncated negative binomial models were fitted as sensitivity analyses (Supplemental Material S2).

We further performed an exploratory factor analysis on the symptom variables to form symptom groups. We determined the number of factors by scree test and parallel analysis. Factors were computed using a maximum likelihood approach and rotated with the oblimin method. Factor scores were estimated using the regression method and interpreted as relative symptom burden.

All statistical analyses were performed in R version 4.5.1. Survival analyses for duration of symptoms were conducted using the *survival*^20^ package. Ordinal logistic regression was performed with the *MASS*^21^ package. Factor analysis was performed using the *psych*^22^ package.

## Results

Of all 128,748 invited DigiHero participants 50,649 (39.3%) responded to a survey conducted in September 2025 assessing infections and subsequent long-lasting symptoms between September 2024 and August 2025. 3,184 participants were excluded from further analyses due to missing data on one or more variables (sex, age, infection during the study period). Of respondents, 24,621 (48.6%) reported no infection, 18,734 (37.0%) reported an infection without following long-lasting symptoms and 2,362 (4.7%) reported they did not know or could not remember an infection. 4,360 (8.6%) participants reported having had a respiratory infection and subsequent long-lasting symptoms and were invited to a follow-up survey. From this group, 3,239 (74.3%) participants responded in the follow-up (comparison of age and sex distribution of those invited and respondents of the two follow-up versions in Supplementary Table S1). Of those, 438 were excluded from further analyses because they reported their prolonged symptoms were not in relation to the infection (self-assessment), their infection was less than 12 weeks ago or did not answer this question, they reported multiple pathogens or inconsistent tests results or they did not answer if they already experienced prior PAIS episodes (Flowchart in Supplement Figure S2). Thus, the sample used in further analyses consisted of 2,801 participants.

Overall, 1,609 (57.4%) reported having experienced their first episode of long-lasting symptoms following an infection, while 1,192 (42.6%) had experienced at least one PAIS episode before. Of the latter, 303 (25.4%) reported that they had recovered from their preceding PAIS episode at the time of the new infection resulting in PAIS, 308 (25.8%) reported their symptoms were still ongoing and for 581 (48.7%) this was not assessed. Participants with a recurring PAIS episode reported they had experienced a median of 2 prior PAIS episodes (1: 23.4%, 2–3: 47.2%, 4 or more: 29.4%).

With respect to the distribution of sex, age groups, causing pathogen (if known) and prior infections and vaccinations, we observed only minor differences between those experiencing their first PAIS episode and those for whom PAIS was recurring. Most notably, those with a recurring episode were more often female (74.4% vs. 66.2%) and had reported a higher number of previous SARS-CoV-2 infections on average (mean 2.17 vs. 1.70 infections, Table 1).

**Table 1:** Characteristics of the study population stratified by first and recurring PAIS episodes. N (%) for categorical variables and mean (standard deviation (SD)) for continuous variables.

		Total	first PAIS	recurring PAIS, overall	recurring PAIS, recovered before new infection	recurring PAIS, not recovered before new infection	recurring PAIS, recovery not assessed
Total, *N* (%)		2801 (100)	1609 (57.4)	1192 (42.6)	303 (10.8)	308 (11.0)	581 (20.7)
	N (%)						
Sex	Male	843 (30.1)	540 (33.6)	303 (25.4)	87 (28.7)	79 (25.6)	137 (23.6)
	Female	1,952 (69.7)	1,065 (66.2)	887 (74.4)	215 (71.0)	229 (74.4)	443 (76.2)
	Other	6 (0.2)	4 (0.2)	2 (0.2)	1 (0.3)	0 (0.0)	1 (0.2)
Age	20–29	93 (3.3)	62 (3.9)	31 (2.6)	11 (3.6)	12 (3.9)	8 (1.4)
	30–39	284 (10.1)	180 (11.2)	104 (8.7)	22 (7.3)	20 (6.5)	62 (10.7)
	40–49	469 (16.7)	266 (16.5)	203 (17.0)	50 (16.5)	48 (15.6)	105 (18.1)
	50–59	697 (24.9)	371 (23.1)	326 (27.3)	87 (28.7)	80 (26.0)	159 (27.4)
	60–69	836 (29.8)	478 (29.7)	358 (30.0)	94 (31.0)	91 (29.5)	173 (29.8)
	70+	422 (15.1)	252 (15.7)	170 (14.3)	39 (12.9)	57 (18.5)	74 (12.7)
Pathogen	SARS-CoV-2	482 (17.2)	245 (15.2)	237 (19.9)	39 (12.9)	72 (23.4)	126 (21.7)
	Influenza	103 (3.7)	62 (3.9)	41 (3.4)	12 (4.0)	8 (2.6)	21 (3.6)
	RSV	37 (1.3)	20 (1.2)	17 (1.4)	3 (1.0)	8 (2.6)	6 (1.0)
	Other pathogen	63 (2.2)	40 (2.5)	23 (1.9)	9 (3.0)	5 (1.6)	9 (1.5)
	Unknown pathogen	2,116 (75.5)	1,242 (77.2)	874 (73.3)	240 (79.2)	215 (69.8)	419 (72.1)
Severity of acute infection	Very mild	35 (1.2)	26 (1.6)	9 (0.8)	2 (0.7)	2 (0.6)	5 (0.9)
	Mild	493 (17.6)	302 (18.8)	191 (16.0)	46 (15.2)	48 (15.6)	97 (16.7)
	Moderate	1,710 (61.0)	992 (61.7)	718 (60.2)	190 (62.7)	180 (58.4)	348 (59.9)
	Severe	488 (17.4)	243 (15.1)	245 (20.6)	61 (20.1)	66 (21.4)	118 (20.3)
	Very severe	62 (2.2)	35 (2.2)	27 (2.3)	3 (1.0)	11 (3.6)	13 (2.2)
	Missing	13 (0.5)	11 (0.7)	2 (0.2)	1 (0.3)	1 (0.3)	0 (0.0)
Influenza vaccination in winter 2024/2025	Yes	734 (26.2)	380 (23.6)	354 (29.7)	94 (31.0)	97 (31.5)	163 (28.1)
	No	883 (31.5)	461 (28.7)	422 (35.4)	94 (31.0)	127 (41.2)	201 (34.6)
	No answer	1,184 (42.3)	768 (47.7)	416 (34.9)	115 (38.0)	84 (27.3)	217 (37.3)
	Mean (SD)						
Number of preceding SARS-CoV-2 infections		1.90 (1.69)	1.70 (1.76)	2.17 (1.57)	2.14 (1.71)	2.18 (1.50)	2.18 (1.52)
Number of preceding vaccinations against SARS-CoV-2		3.23 (1.22)	3.26 (1.20)	3.20 (1.24)	3.26 (1.19)	3.13 (1.29)	3.21 (1.24)

**Fig. 1:**
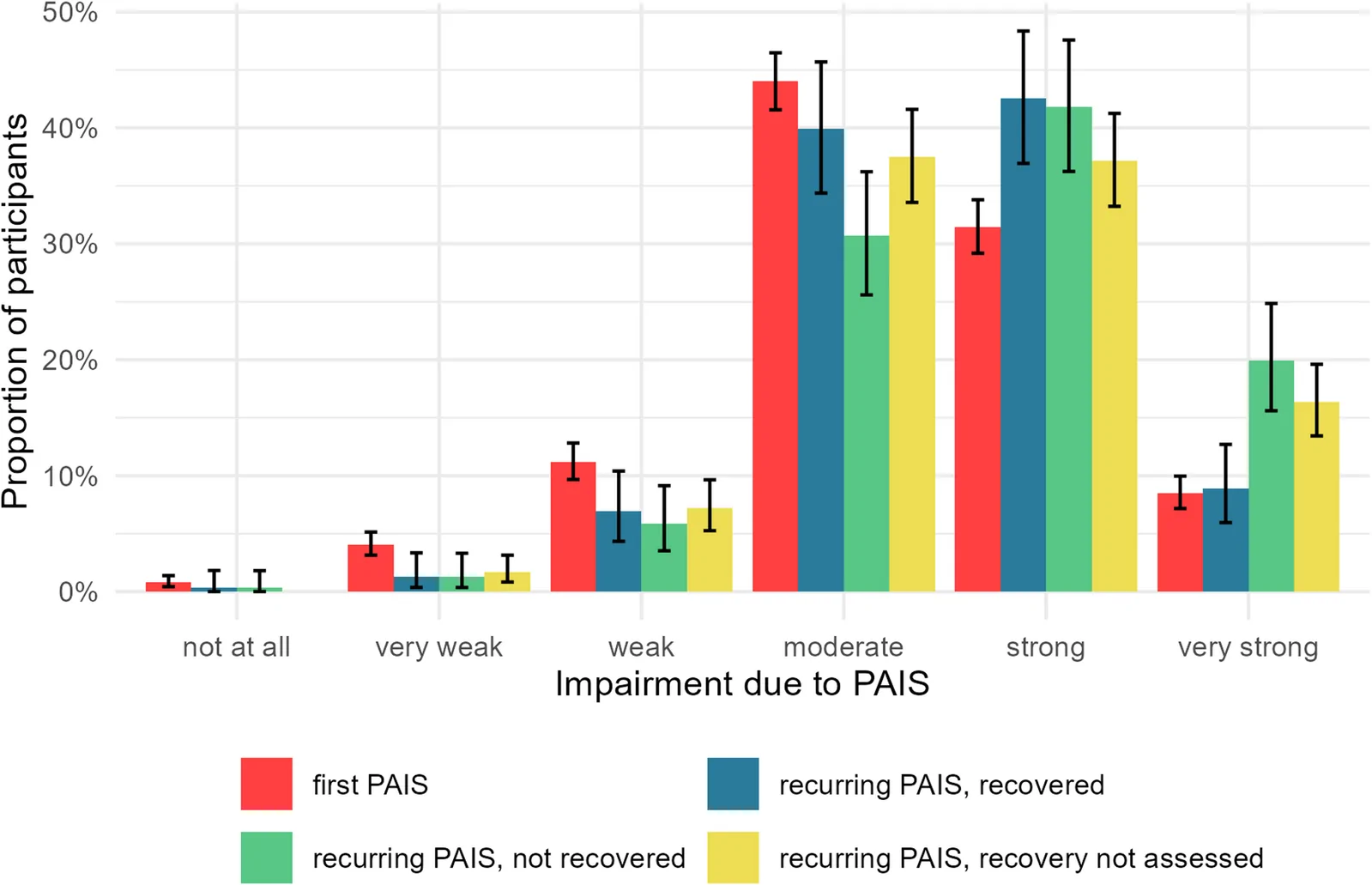
Proportions and 95%-CI of reported impairment due to long-lasting symptoms compared between first and recurring PAIS episodes (*N* = 2,792; 9 participants excluded due to missing data).

With regard to self-reported impairment due to the long-lasting symptoms, we observed that participants who experienced their first PAIS episode reported a lower impairment on average, with 84.0% [95%-CI: 82.1%; 85.7%] reporting moderate to very strong impairment compared to 92.5% [95%-CI: 88.9%; 95.2%] among recurring PAIS cases that had not yet recovered, and 91.4% [95%-CI: 87.8%; 94.3%] of previously recovered recurring PAIS cases (Fig. 1). In a multivariable ordinal logistic regression adjusted for age, sex, pathogen, number of prior SARS-CoV-2 infections and vaccination status for SARS-CoV-2 and influenza, we observed higher odds of having a higher level of impairment (Odds Ratio (OR) = 1.5 [95%-CI: 1.1; 1.9]) for recurring PAIS cases that had previously recovered compared to first PAIS cases and for those recurring PAIS cases that had not yet recovered (OR = 2.0 [95%-CI: 1.5; 2.6]). Consistently, the group with unknown recovery status was between those with recurring PAIS after recovery and those not recovered before (Supplementary Table S2).

For those recurring PAIS cases that rated their impairment of the most recent episode compared to their first episode (*N* = 601), 42.5% [95%-CI: 38.6%; 46.7%] reported it was the same. 25.6% [95%-CI: 22.2%; 29.3%] reported the current episode was less or much less severe and 31.7% [95%-CI: 28.1%; 35.7%] reported it was more or much more severe. Proportions were broadly similar between those that had recovered from an earlier episode and those that had not recovered (Supplementary Figure S3).

Participants that experienced their first PAIS episode reported on average 6.4 [95%-CI: 6.2; 6.6] symptoms (of a list with 24 common PAIS symptoms) and those with recurring PAIS that had recovered before the new episode reported on average 7.0 [95%-CI: 6.5; 7.5] symptoms. In comparison, those with recurring PAIS who had not yet recovered from the last episode reported a substantially higher number of symptoms (mean: 8.9 [95%-CI: 8.3; 9.5], Supplementary Figure S4). These observations remained stable after adjusting for potential confounders in the linear regression. Compared to first PAIS cases, recurring but not recovered PAIS cases were estimated to report 2.0 [95%-CI: 1.5; 3.0] additional symptoms. There was no difference however between first and recurring but previously recovered PAIS episodes. Again, those for whom recovery was not assessed were in between recurring recovered and non-recovered cases (Supplementary Table S2).

**Fig. 2:**
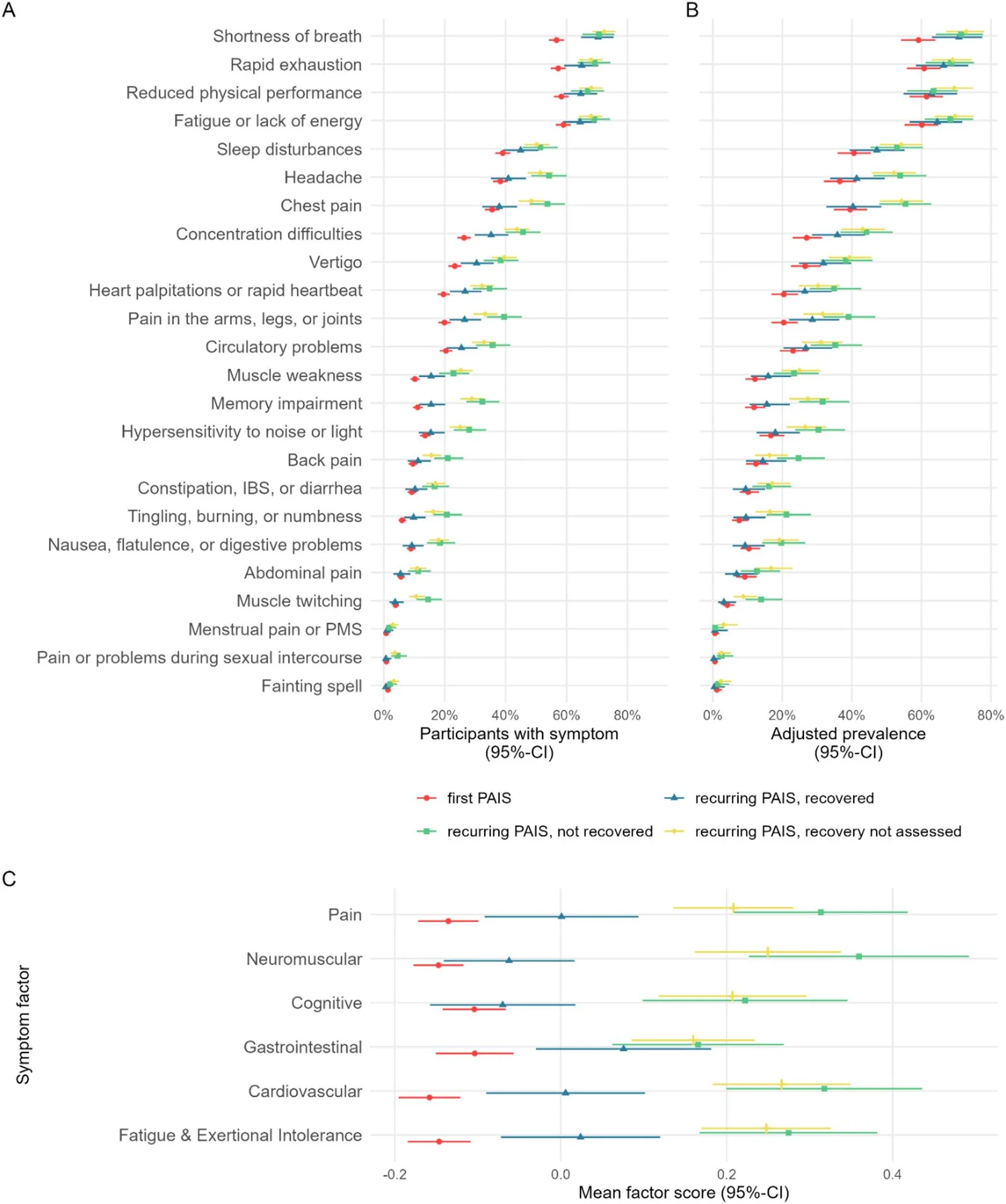
(**A**) Proportions and 95%-CIs of participants reporting specific symptoms (*N* = 2,801), (**B**) symptom prevalences adjusted for age, sex, pathogen, severity of the acute infection, number of previous SARS-CoV-2 vaccinations, vaccination against influenza in the last winter season, and number of previous SARS-CoV-2 infections (complete case analysis, *N* = 2,724), and (**C**) mean factor scores for six symptom groups (*N* = 2,801) compared between first and recurring PAIS episodes. Factor scores are centred on the sample mean (0), with higher values reflecting greater relative symptom burden compared to the overall sample.

With regard to proportions of participants reporting individual symptoms, there were substantial similarities for all four groups. The relative ranking of individual symptoms was broadly similar, with shortness of breath, rapid exhaustion, reduced physical performance, and fatigue among the most frequently reported symptoms. However, differences in absolute symptom prevalence were apparent for several symptoms, particularly those of intermediate prevalence (e.g. muscle twitching, noise/light sensitivity, memory impairment, chest pain and headache). In these instances, participants with first PAIS and those with recurring PAIS after recovery generally reported lower prevalences than participants with recurring PAIS who had not recovered or whose recovery status had not been assessed. For less prevalent symptoms, estimates overlapped considerably across groups (Fig. 2A).

After adjustment, the overall pattern was the same, but group differences reduced. While symptom profiles were similar across groups, first PAIS episodes and recurring but previously recovered PAIS cases displayed lower prevalences compared to the non-recovered and unknown status recurring PAIS cases for almost all symptoms (Fig. 2B). This observation was also consistent with the aforementioned differences in the number of reported symptoms (Supplementary Figure S4). Largest differences between first PAIS cases and recurring, non-recovered PAIS cases in adjusted prevalences were observed for memory impairment, joint pain, headache and concentration problems while there were no differences for rapid exhaustion, reduced physical performance or fatigue.

An exploratory factor analysis on the symptom variables revealed six symptom groups (cardiovascular, gastrointestinal, cognitive, neuromuscular symptoms, fatigue/exertional intolerance, and pain, Supplementary Table S3). Comparing mean factor scores across the four groups showed a similar, but more expressed pattern compared to the individual symptom variables. Individuals experiencing their first PAIS episode displayed the lowest mean factor scores for all six symptom groups. Recurring but previously recovered PAIS cases expressed higher factor scores for fatigue/exertional intolerance, pain, cardiovascular, and gastrointestinal symptoms. Non-recovered recurring cases and PAIS cases for whom recovery status was not assessed displayed the highest factor scores across all six symptom groups (Fig. 2C).

**Fig. 3:**
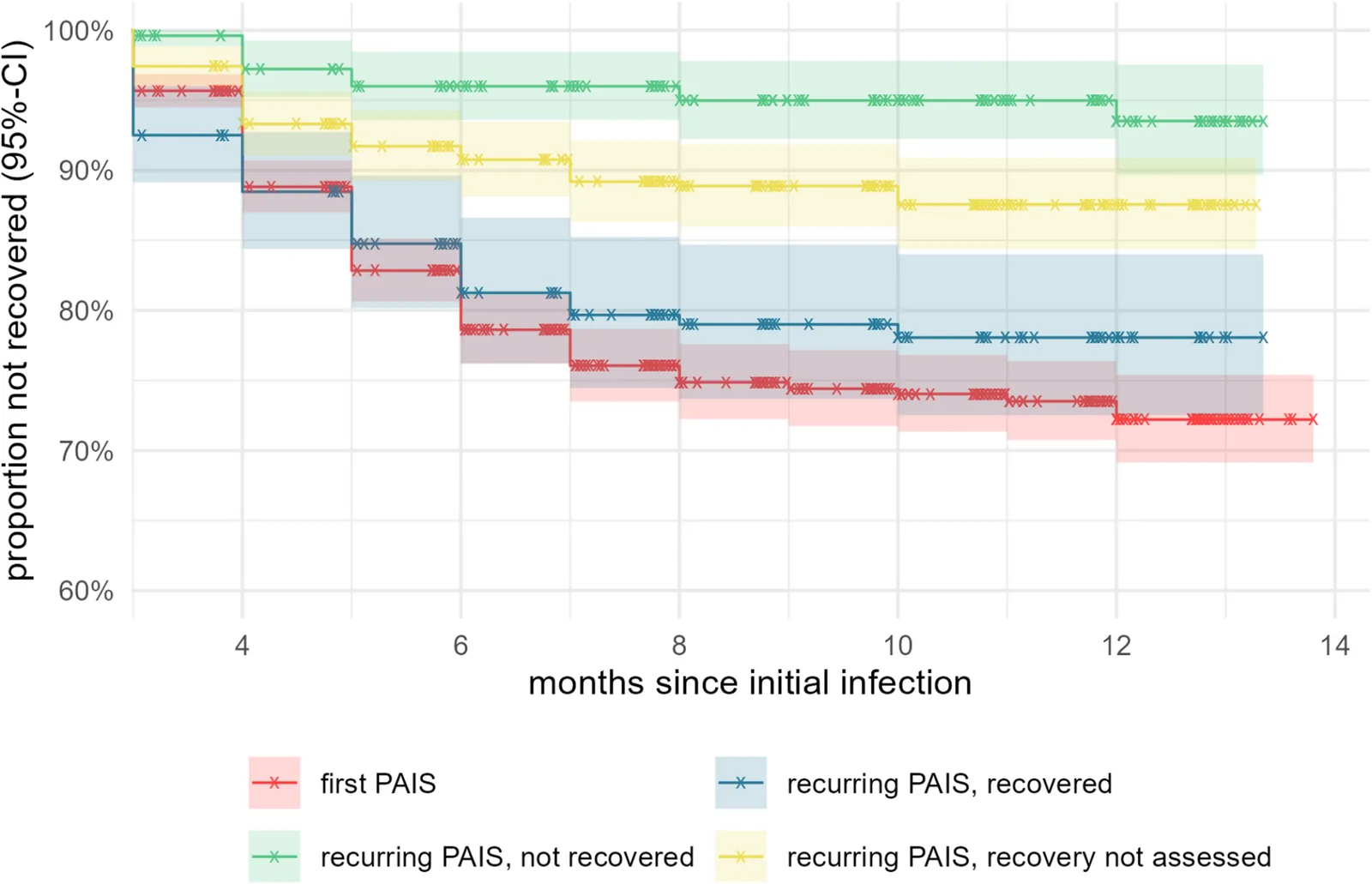
Duration of long-lasting symptoms (Kaplan-Meier curves and 95%-CIs) compared between first and recurring PAIS episodes (*N* = 2,106; 695 participants were excluded for missing data on month of infection or month of recovery from long-lasting symptoms.).

For duration of symptoms, we observed the lowest 12-month recovery rate for participants with a recurring PAIS episode who had not recovered before the new infection with only 6.5% [95%-CI: 2.4; 10.3] estimated recovery. Of those who recovered from a prior PAIS episode before the new infection, 21.9% [95%-CI: 16.0; 27.5] and of those with a first PAIS episode 27.8% [95%-CI: 24.6; 30.9] were estimated to have recovered after 12 months. The estimated recovery after 12 months of those recurring PAIS cases for whom prior recovery status was not assessed was between those recurring cases with and without prior recovery (12.4% [95%-CI: 9.1; 15.6], Fig. 3). This observation was also seen in the adjusted Cox regression. Recurring, not previously recovered cases had a significantly lower hazard of recovery (Hazard Ratio (HR): 0.2 [95%-CI: 0.1; 0.4]) than recurring PAIS cases that had recovered (HR: 0.9 [95%-CI: 0.7; 1.3]) compared to first PAIS cases (Supplementary Table S2).

## Discussion

Among 2,801 DigiHero participants classified as PAIS cases who reported a respiratory infection between September 2024 and August 2025 who also completed a follow-up questionnaire, recurring PAIS was common and was associated with a greater subsequent symptom burden than first PAIS when not fully recovered from a previous PAIS episode. Participants with recurring PAIS reported higher impairment, more symptoms, and a lower rate of recovery, with the most pronounced burden observed among those who reported that they had not recovered from their previous PAIS episode before the subsequent infection. Across the four groups, the relative ordering of individual symptoms was broadly similar, whereas differences were apparent in their absolute prevalence and in the six symptom-domain scores. Recurring PAIS who were not fully recovered from the previous episode reported higher symptom prevalences across all symptom domains. Adjusted analyses largely reproduced these descriptive patterns.

The importance of previous recovery status represents a central finding of this study. Previous research on SARS-CoV-2 reinfections has shown that repeated infections may add to the cumulative risk and burden of post-acute sequelae^13,23^. However, these studies have generally not distinguished between a new PAIS episode after recovery, an exacerbation of residual symptoms, and the continuation of an existing condition across a subsequent infection. In our study, participants who had not recovered before the subsequent infection consistently showed the greatest impairment, highest symptom counts, and lowest estimated recovery. This group may therefore include participants for whom the subsequent infection exacerbated an ongoing condition or added new symptoms to an existing symptom burden. By contrast, participants who reported having recovered before the subsequent infection generally showed less pronounced differences from participants with first PAIS. Despite the observational and self-reported nature of the data, these findings suggest that previous recovery status may be an important characteristic to account for in future studies of repeated infections and recurring PAIS.

While we observed a higher impairment for recurring PAIS cases than for first episodes, interestingly, most recurring PAIS cases assessed their impairment as similar to their first episode. There are several possible explanations. Recall bias may have led participants to assess their prior symptoms similar to the most recent episode. Also, individuals with infections during recovery from a PAIS episode may have experienced a longer first episode than those experiencing their first episode at the time of this study. According to Tran et al., longer symptom duration leads to an increasing individual impairment^24^. Lastly, it might be that recurring PAIS cases already experienced a higher level of symptom burden in their first episode. This may point to the fact that some individuals are more susceptible to severe PAIS and resurgences of the disease in a similar form after a new infection. This question grants the need for further longitudinal research on recurring PAIS and its risk factors. Though, it should be noted that the pathogen of previous infections followed by PAIS symptoms was not collected in this study. It is possible that participants with recurring PAIS previously experienced symptoms following a different infection than the pathogen of the current episode.

Compared to earlier research in the same cohort, the observed number of symptoms, symptom patterns and recovery were largely similar to those reported for post-COVID cases during the pandemic^12,18^, thus adding to the growing evidence of PAIS as a general sequela of infections. While the observed duration of symptoms in this study is also similar to recovery rates observed for post-COVID in other samples^24^, it was slower than recovery rates observed in some studies using different case definitions or an earlier start of the observation period^5,25^.

There are some limitations to our study. This analysis solely relies on retrospective, self-reported assessments of infections and subsequent symptoms. There might be an influence of recall bias and selective reporting. For example, we might underestimate the number of PAIS cases with a low level of impairment as these might not consider their symptoms as relevant. This influence of recall bias might also be relevant for the reported symptom duration. Cases in which PAIS started early in our study period have a longer recall time and are thus likely to report less precisely. Also, missing information on the date of infection or the time of recovery might have introduced further selection. Additionally, our study likely underrepresents very severe or hospitalised PAIS cases as these individuals might be too impaired to participate.

Our study also did not include prospective recording of infections and systematic testing of pathogens, leading to under- and overreporting of infections in the study period and a high proportion of infections with an unknown underlying pathogen. Most participants reported they did not perform a test (75.5%). Those that reported a test result most often reported SARS-CoV-2 or influenza infections. Most likely, this reflects the availability of rapid self-tests for these pathogens. In an earlier analysis we could show however that PAIS cases resulting from known SARS-CoV-2 and influenza infections were similar regarding impairment, symptom profiles and duration^19^. According to national genomic surveillance data, SARS-CoV-2 accounted for only a minority of respiratory infections during our study period; most were influenza cases^26^.

The DigiHero cohort, while implementing a population-based sampling approach, is prone to selective participation, posing another limitation. This is also caused by the digital design of the study, disregarding the very old or those without access to the internet. Also, the study design with a follow-up of only those reporting long-lasting symptoms, did not allow to compare PAIS cases to a comparator group without these symptoms. Lastly, our assessment of the recovery from prior PAIS episodes at the time of the recent infection was only binary and included partial recovery from symptoms into the persistent group. This might lead to an underestimation of the differences between recovered and persistent recurring PAIS cases. As PAIS symptoms can also resurge irrespective of a new infection and we did not require a minimal symptom-free period for the definition of recovery prior to infection, we could also not differentiate a new episode and the resurgence of an ongoing episode. Therefore, we relied on self-attribution of recovery and new episodes. Also, heterogeneity in the duration of the time since the last episode among recovered could not be accounted for, potentially leading to bias.

## Conclusions

To conclude, we could show that a high proportion of PAIS cases in the period of September 2024 to August 2025 in the DigiHero cohort were recurring episodes of individuals that had already experienced a prior PAIS episode. These recurring PAIS cases displayed a higher level of impairment due to their long-lasting symptoms, a higher number and longer duration of symptoms. At the same time, those who recovered from their previous episode had similar symptoms to those experiencing PAIS for the first time. These findings suggest that recovery status before an infection may be clinically relevant for the expected course of PAIS after a new infection. Participants who had recovered from a previous episode showed outcomes more similar to those experiencing PAIS for the first time, whereas a new infection during ongoing PAIS was associated with a substantially greater symptom burden, impairment, and prolonged recovery. Information on previous PAIS episodes and current recovery status could thus support prognostic assessment, clinical monitoring, and treatment planning. The findings further suggest that individuals with ongoing PAIS may represent a particularly vulnerable group in whom prevention of subsequent respiratory infections deserves greater attention. Future research should identify risk factors for PAIS recurrence and evaluate targeted preventive and early therapeutic strategies.

## Supplementary Information

Below is the link to the electronic supplementary material.

Supplementary Material 1

## Data Availability

Data used in this study is available upon request to the corresponding author and after approval by the Use and Access Committee of the DigiHero study (digihero-studie@uk-halle.de).

## Abbreviations

COVID-19: Corona virus disease 2019
OR: Odds Ratio
PAIS: Post-acute infection syndromes
RSV: Respiratory syncytial virus
SARS-CoV-2: Severe acute respiratory syndrome corona virus 2
SD: Standard deviation
95%-CI: 95% confidence interval
