# Supplementary material for: Comparison of new and recurring post-acute infection syndrome – results from a German population-based cohort
Source: Sci Rep. 2026 Sep 12;16:28492. doi: 10.1038/s41598-026-70451-3 (PMC13569484; doi:10.1038/s41598-026-70451-3)
Supplement: Supplementary file 1 [file 41598_2026_70451_MOESM1_ESM.pdf]

# Supplementary Materials

## Supplement S1: Questionnaires

**Initial Questionnaire (Translated from German)**

**Have you had symptoms since September 2024 that appeared after a respiratory infection and persisted for 12 or more weeks after the infection?**

Respiratory infections include, for example, influenza, coronavirus (SARS-CoV-2 and other coronaviruses), RSV or other illnesses with cold symptoms such as cough, runny nose, sore throat or fever.

- ☐ I didn’t have a respiratory infection since September 2024.
- ☐ I had a respiratory infection since September 2024, but no symptoms that have newly appeared or increased since then and persisted for 12 or more weeks after the infection.
- ☐ Yes, I had a respiratory infection since September 2024 and symptoms that have newly occurred or increased since then and persisted for 12 or more weeks after the infection.
- ☐ I don't know or I can't remember.
- ☐ I don't want to answer this question.

**Follow Up Questionnaire (Translated from German)**

**In the last survey, you indicated that since September 2024, you have had a respiratory infection and symptoms that subsequently appeared or worsened and persisted for 12 or more weeks after the infection.**

**Did you perceive these symptoms to be related to the respiratory infection?**

- ☐ Yes
- ☐ No
- ☐ Don’t know
- ☐ My respiratory infection was less than 12 weeks ago.

**[Only continue, if Yes or I don’t know]**

**When did you have the respiratory infection that led to your long-lasting symptoms?**

Month / year

**How would you describe the overall severity of your acute illness?**

- ☐ Very mild
- ☐ Mild
- ☐ Moderate
- ☐ Severe
- ☐ Very severe

**Did you perform a rapid self-test during this infection to detect the pathogen?**

- ☐ Yes
- ☐ No

**[If Yes:] Which pathogen was detected in the self-test?**

- ☐ Influenza (flu)
- ☐ SARS-CoV-2
- ☐ RSV (Respiratory syncytial virus)
- ☐ Another pathogen: _________________
- ☐ No pathogen was detected.
- ☐ Don’t know

**Did a doctor take a swab from you to detect the pathogen?**

- ☐ Yes
- ☐ No

**[If yes:] Which pathogen was detected in the swab test by a doctor?**

- ☐ Influenza (flu)
- ☐ SARS-CoV-2
- ☐ RSV (Respiratory syncytial virus)
- ☐ Another pathogen: _________________
- ☐ No pathogen was detected.
- ☐ Don’t know

**Which of the following symptoms did you experience 12 or more weeks after the acute infection?**

| | Symptom was not present | Symptom was present but was/is not related to the acute respiratory infection | Symptom was present and was/is related to the acute respiratory infection |
|---|---|---|---|
| Abdominal pain | ☐ | ☐ | ☐ |
| Back pain | ☐ | ☐ | ☐ |
| Pain in the arms, legs, or joints (knees, hips, etc.) | ☐ | ☐ | ☐ |
| Menstrual pain or PMS (Premenstrual syndrome) | ☐ | ☐ | ☐ |
| Pain or problems during sexual intercourse | ☐ | ☐ | ☐ |
| Headache | ☐ | ☐ | ☐ |
| Chest pain | ☐ | ☐ | ☐ |
| Vertigo | ☐ | ☐ | ☐ |
| Fainting spell | ☐ | ☐ | ☐ |
| Heart palpitations or rapid heartbeat | ☐ | ☐ | ☐ |
| Shortness of breath | ☐ | ☐ | ☐ |
| Constipation, irritable bowel syndrome, or diarrhea | ☐ | ☐ | ☐ |
| Nausea, flatulence, or digestive problems | ☐ | ☐ | ☐ |
| Difficulty falling asleep or staying asleep, or increased sleep | ☐ | ☐ | ☐ |
| Fatigue or lack of energy | ☐ | ☐ | ☐ |
| Rapid exhaustion | ☐ | ☐ | ☐ |
| Concentration difficulties | ☐ | ☐ | ☐ |
| Memory impairment | ☐ | ☐ | ☐ |
| Reduced physical performance | ☐ | ☐ | ☐ |
| Hypersensitivity to noise or light | ☐ | ☐ | ☐ |
| Circulatory problems such as dizziness, | ☐ | ☐ | ☐ |

| | | | |
|---|---|---|---|
| **weakness, or palpitations when standing up** | | | |
| **Sensory disturbances such as tingling sensations, burning sensations, or numbness** | ☐ | ☐ | ☐ |
| **Muscle twitching** | ☐ | ☐ | ☐ |
| **Muscle weakness** | ☐ | ☐ | ☐ |
| **Other symptoms** | ☐ | ☐ | ☐ |

**How much did the symptoms affect you when they were at their worst?**

- ☐ Not at all
- ☐ Very little
- ☐ A little
- ☐ Moderately
- ☐ Strongly
- ☐ Very strongly

**Do you still have at least one of the above symptoms?**

- ☐ Yes
- ☐ No

**[If Yes:] How long did you have persistent symptoms after your infection?**

Month / year

**How many times have you knowingly had COVID-19 (SARS-CoV-2) so far?**

______________

**How many COVID-19 (SARS-CoV-2) vaccinations have you had in total so far?**

______________

**Did you receive a vaccination against influenza (the flu) in autumn/winter 2024/2025?**

- ☐ Yes
- ☐ No

**Did you already experience any late symptoms or long-term effects following an acute respiratory infection before September 2024?**

- ☐ Yes
- ☐ No

**Had these earlier symptoms already subsided before the new infection?**

- ☐ Yes
- ☐ No

**How often have you experienced long-lasting symptoms following an infection?**

_____________ times

**How did/do the long-lasting symptoms following the current infection compare with those following the first infection, after which the long-lasting symptoms first appeared?**

- ☐ Much less severe
- ☐ Less severe
- ☐ About the same
- ☐ More severe
- ☐ Much more severe

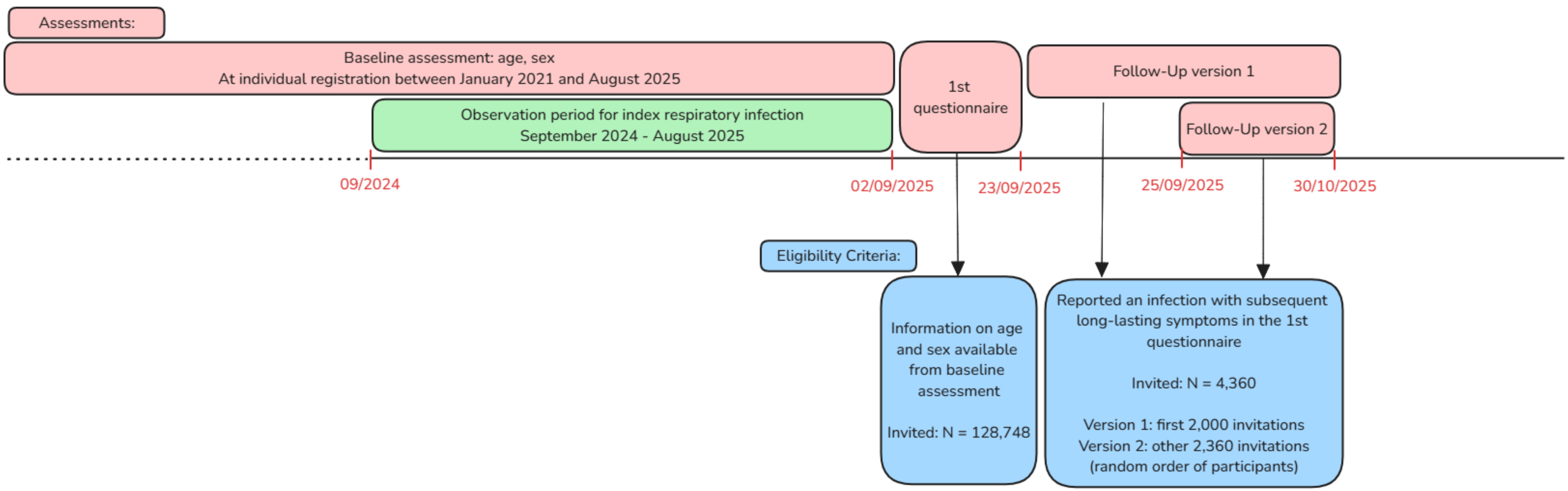


*Figure S1: Timeline of assessments and eligibility criteria.*

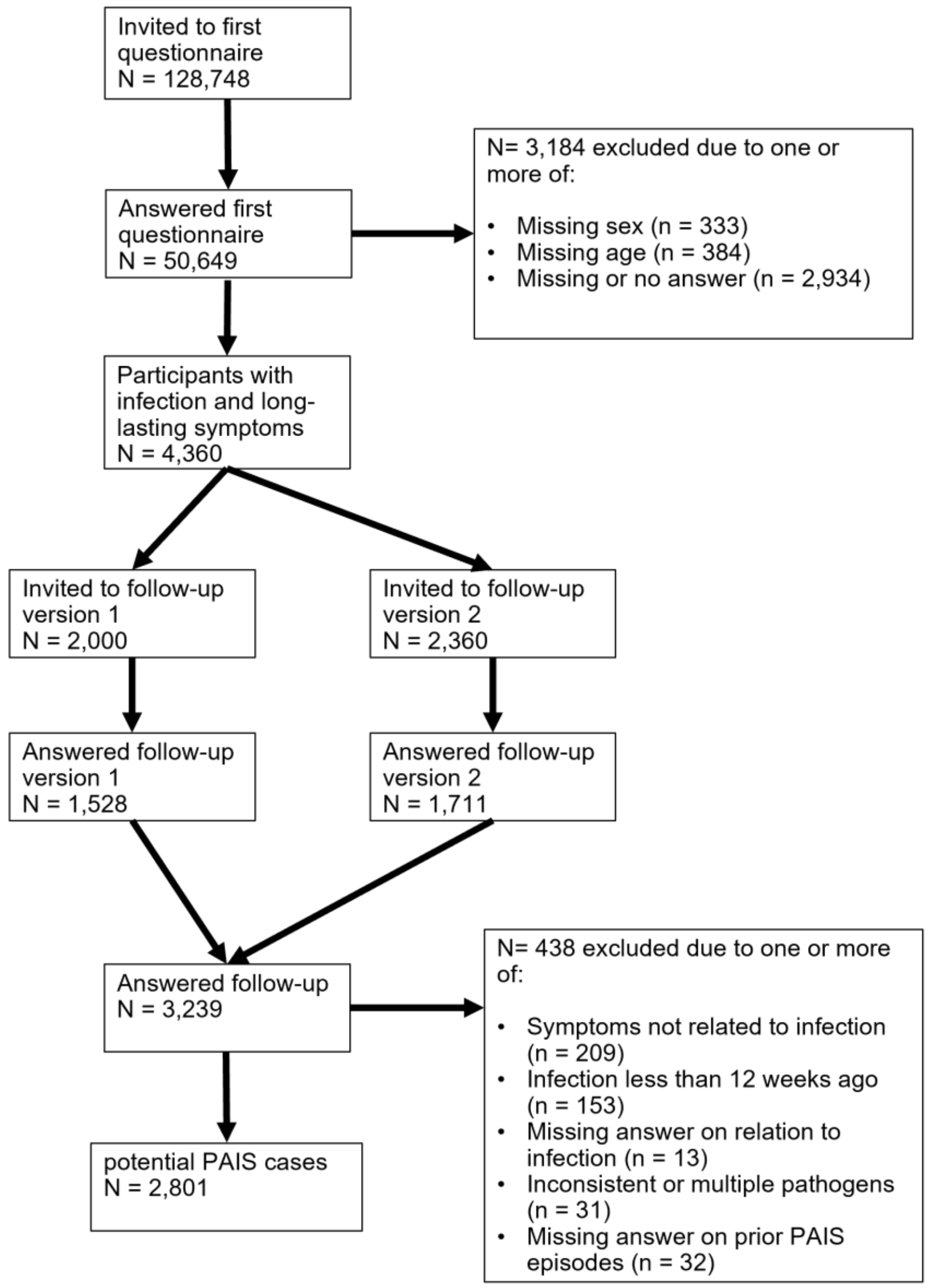


*Figure S2: Flowchart of the study population. After 2,000 invitations to the follow-up survey, additional questions on the recovery status at the time of the new infection and on the impairment due to the recurring episode compared to the first PAIS episode were added in a second version of the questionnaire.*

*Table S1: Comparison of age, sex and PAIS episode of participants invited and respondents of follow-up versions*

| | | Invited to follow-up | | Answered follow-up | |
|---|---|---|---|---|---|
| | | **Version 1** | **Version 2** | **Version 1** | **Version 2** |
| **Total, N (%)** | | 2000 (45.9) | 2360 (54.1) | 1528 (47.2) | 1711 (52.8) |
| **Sex** | male | 615 (31.0) | 745 (31.9) | 459 (30.0) | 534 (31.2) |
| | female | 1366 (68.8) | 1581 (67.7) | 1068 (69.9) | 1171 (68.4) |
| | other | 4 (0.2) | 8 (0.3) | 1 (0.1) | 6 (0.4) |
| **Age** | 20-29 | 75 (3.8) | 103 (4.4) | 39 (2.6) | 70 (4.1) |
| | 30-39 | 256 (12.9) | 241 (10.3) | 180 (11.8) | 152 (8.9) |
| | 40-49 | 310 (15.7) | 395 (16.9) | 236 (15.4) | 284 (16.6) |
| | 50-59 | 470 (23.8) | 554 (23.8) | 391 (25.6) | 395 (23.1) |
| | 60-69 | 550 (27.8) | 668 (28.7) | 450 (29.5) | 529 (30.9) |
| | 70+ | 317 (16.0) | 370 (15.9) | 232 (15.2) | 281 (16.4) |
| **PAIS before** | first PAIS | --- | --- | 459 (30.0) | 534 (31.2) |
| | recurring PAIS | --- | --- | 1068 (69.9) | 1171 (68.4) |

*Table S2: Results from adjusted regressions.*

| | Impairment due to PAIS (OR [95%-CI]) | | Number of Symptoms (Estimate [95%-CI]) | Duration of symptoms (Hazard Ratio [95%-CI]) |
|---|---|---|---|---|
| regression model | multivariable ordinal logistic regression | | multivariable linear regression | Cox proportional hazards regression |
| N included in the model | 2,718 | | 2,724 | 2,040 |
| **(Intercept)** | low - moderate<br>moderate - high | 0.01 [0.01; 0.02]<br>0.13 [0.07; 0.22] | 10.17 [9.05; 11.30] | |
| **first PAIS vs. recurring PAIS** | | | | |
| first PAIS | *Ref.* | | *Ref.* | *Ref.* |
| recurring PAIS, recovered | 1.47 [1.14; 1.91] | | 0.29 [-0.07; 1.05] | 0.92 [0.67; 1.26] |
| recurring PAIS, not recovered | 1.96 [1.49; 2.59] | | 2.01 [1.45; 2.57] | 0.21 [0.12; 0.37] |
| recurring PAIS, recovery not assessed | 1.49 [1.21; 1.82] | | 1.73 [1.30; 2.17] | 0.46 [0.34; 0.62] |
| **sex** | | | | |
| male | *Ref.* | | *Ref.* | *Ref.* |
| female | 1.69 [1.43; 2.01] | | 0.24 [-0.14; 0.63] | 0.75 [0.60; 0.93] |
| **age (10 years)** | 0.93 [0.87; 0.99] | | -0.08 [-0.22; 0.06] | 0.92 [0.85; 1.00] |
| **pathogen** | | | | |
| SARS-CoV-2 | *Ref.* | | *Ref.* | *Ref.* |
| Influenza | 0.85 [0.53; 1.38] | | 0.03 [-0.92; 0.97] | 1.05 [0.53; 2.08] |
| Unknown/Other pathogen | 0.66 [0.53; 0.83] | | -1.57 [-2.03; -1.11] | 1.38 [1.03; 1.84] |
| **Severity of the acute infection** | | | | |
| Severe | *Ref.* | | *Ref.* | *Ref.* |
| Mild | 0.04 [0.03; 0.05] | | -3.38 [-3.97; -2.80] | 1.13 [0.94; 1.69] |
| Moderate | 0.17 [0.13; 0.21] | | -1.81 [-2.25; -1.37] | 1.26 [0.94; 1.69] |
| **Influenza vaccination in 2024/2025** | | | | |
| No | *Ref.* | | *Ref.* | *Ref.* |
| Yes | 0.79 [0.64; 0.99] | | -0.50 [-0.97; -0.03] | 1.17 [0.88; 1.57] |
| Missing | 0.35 [0.29; 0.43] | | -0.66 [-1.08; -0.25] | 1.49 [1.17; 1.91] |
| **Nr. SARS-CoV-2 infections** | 1.03 [0.98; 1.08] | | 0.11 [0.01; 0.21] | 0.93 [0.86; 1.01] |
| **Nr. SARS-CoV-2 vaccinations** | 1.03 [0.96; 1.10] | | -0.10 [-0.25; 0.06] | 1.04 [0.95; 1.13] |

## Supplement S2: Regression Diagnostics

Multivariable regression models were used to assess whether the descriptively observed differences between PAIS groups persisted after adjustment for age, sex, pathogen, severity of the preceding acute infection, number of COVID-19 vaccinations, influenza vaccination status, and number of previous COVID-19 infections.

For all three models, RSV was combined with other or unknown pathogens and severity of the preceding acute infection was grouped into three categories (mild, moderate and severe) because of small cell counts in the original categories. Additionally, age was divided by 10 to be interpreted as age in 10 years for better interpretability of the coefficients. Further, 13 participants were excluded due to missing information of severity of the preceding acute infection and analyses were restricted to participants with male or female sex, excluding 6 participants with diverse sex. Also, 42 participants were excluded due to missing information on the number of previous COVID-vaccinations and 27 participants were excluded for missing information on the number of previous COVID-infections. Due to a high number of missing information on the administration of an influence vaccination in the last winter season, this was included as a separate category. This procedure resulted in a sample of 2,724 participants. For all three regression models, we conducted a complete case analysis, excluding all participants with relevant missing outcome information:

- 6 participants with missing impairment for the ordinal logistic regression (N = 2,718)
- 684 participants with missing data on month of infection or month of recovery from long-lasting symptoms in the Cox proportional hazards model (N = 2040)

Model diagnostics and sensitivity analyses were conducted to assess whether these estimates were materially affected by violations of the respective model assumptions.

**Impairment: ordinal logistic regression**

Self-reported impairment was combined into three ordered categories: low, moderate, and high impairment. The analytical sample included 347 participants with low, 1,106 with moderate, and 1,265 with high impairment.

The proportional-odds assumption was assessed using likelihood-ratio tests comparing the proportional-odds model with models allowing threshold-specific effects for each model term. There was no evidence that the assumption was violated for the PAIS-group variable as a whole (p=0.649). Evidence of non-proportional effects was observed for severity of the preceding acute infection (p<0.001) and, to a lesser extent, sex (p=0.044). There was no evidence of non-proportional effects was found for age, pathogen, vaccination variables, or the number of previous COVID-19 infections.

We therefore fitted a partial proportional-odds model as a sensitivity analysis, allowing the effects of acute infection severity and sex to vary between the two cumulative outcome thresholds while retaining proportional effects for the PAIS groups and the remaining covariates. Compared with participants with first PAIS, the proportional-odds model yielded odds ratios of 1.47 [95%-CI: 1.14; 1.91] for recurring PAIS after previous recovery, 1.96 [95%-CI: 1.49; 2.59] for recurring PAIS without previous recovery, and 1.49 [95%-CI: 1.21; 1.82] for recurring PAIS with recovery status not assessed. The corresponding estimates from the partial proportional-odds model were 1.47 [95%-CI: 1.14; 1.91], 1.97 [95%-CI: 1.49; 2.60], and 1.49 [95%-CI: 1.22; 1.83], respectively.

Allowing threshold-specific effects for sex and acute infection severity therefore had virtually no effect on the estimated associations between PAIS group and impairment. The likelihood-ratio comparison between the proportional and partial proportional-odds models

yielded $\chi 2 = 24.1$, df=3, and $p<0.001$; the corresponding AIC values were 4506.19 and 4488.12, respectively.

Because the proportional-odds assumption was supported for the exposure of primary interest and the PAIS-group estimates were essentially identical in the more flexible model, the proportional-odds model was retained as the main analysis. The partial proportional-odds model was considered a sensitivity analysis addressing non-proportional effects of adjustment variables that were not themselves the focus of interpretation.

**Number of symptoms: linear regression**

The number of reported symptoms was a positive count ranging from 1 to 24. Among the 2,313 participants included in the model, the mean was 7.20 symptoms (SD 4.51), the median was 7 symptoms (IQR 4–10), and the skewness coefficient was 0.69, indicating moderate right-skewness.

Inspection of residual-versus-fitted and scale–location plots indicated mild heteroscedasticity, while the normal Q–Q plot showed some deviation from residual normality, particularly in the tails. The Breusch–Pagan test yielded $\chi 2=113.27$, df=13, and $p<0.001$. Because formal diagnostic tests are sensitive to relatively small deviations in large samples, the assessment was based on both the graphical and statistical diagnostics. The primary linear regression results were reported with conventional model-based standard errors and confidence intervals. Heteroscedasticity-consistent HC3 standard errors were additionally calculated as a sensitivity analysis.

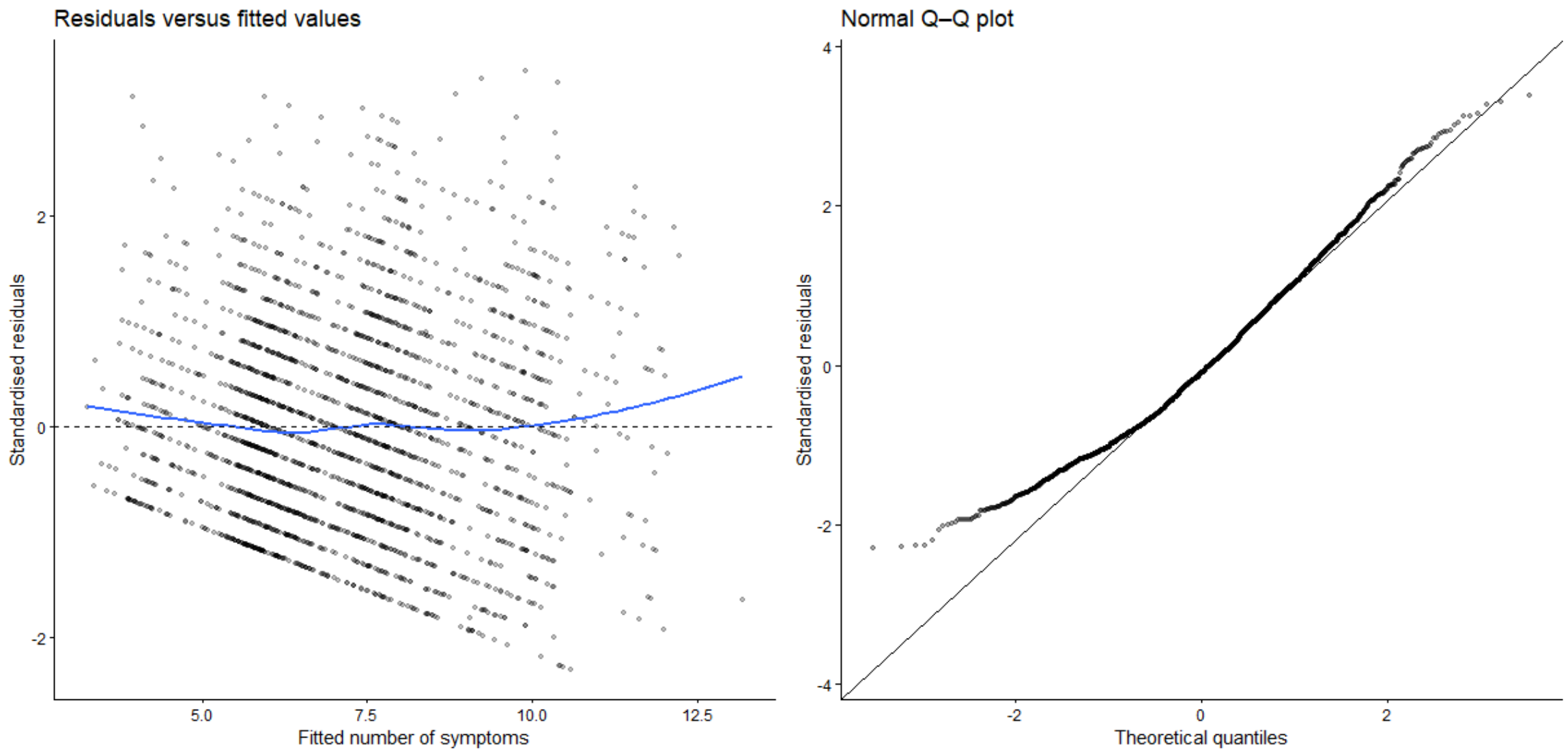


No relevant multicollinearity was observed; all variance inflation factors were close to 1, with a maximum VIF of 1.20.

In the primary linear regression, participants with recurring PAIS after previous recovery reported an estimated 0.49 additional symptoms compared with participants with first PAIS [95%-CI: −0.07; 1.05]. The estimated differences were 2.01 additional symptoms [95%-CI: 1.45; 2.57] among participants who had not recovered before the subsequent infection and 1.73 additional symptoms [95%-CI: 1.30; 2.17] among those whose previous recovery status had not been assessed. Results based on HC3 standard errors were very similar. The corresponding 95% confidence intervals were −0.04 to 1.02, 1.39 to 2.63, and 1.26 to 2.21, respectively, and did not alter the substantive interpretation of any PAIS-group comparison.

Because the outcome was a right-skewed count without zero values, a zero-truncated negative binomial regression was fitted as a sensitivity analysis. Compared with first PAIS, the adjusted mean symptom count was 1.08 times as high among participants with recurring PAIS after previous recovery (mean ratio 1.08 [95%-CI: 0.99; 1.17], 1.31 times as high among participants who had not recovered [95%-CI: 1.21; 1.43], and 1.28 times

as high among participants whose recovery status had not been assessed [95%-CI: 1.20; 1.36].

The absolute mean differences from the primary linear regression, the estimates based on HC3 standard errors, and the relative mean differences from the zero-truncated negative binomial model showed the same ordering and led to the same substantive interpretation. The linear model was retained as the main analysis because its coefficients provide directly interpretable absolute differences in the mean number of symptoms and the alternative inferential specifications did not materially alter the results.

**Duration until recovery: Cox proportional hazards regression**

The Cox regression included 2,040 participants, of whom 377 reported recovery during the observed period and 1,663 remained symptomatic at the time of assessment and were right-censored. Thus, 18.5% of participants experienced the recovery event.

The proportional-hazards assumption was evaluated using scaled Schoenfeld residuals. There was no statistically significant evidence of non-proportional hazards for the PAIS-group variable ($\chi2=5.20$, df=3, p=0.158) or for any individual adjustment variable. The global test also provided no clear evidence against the proportional-hazards assumption ($\chi2=20.07$, df=13, p=0.094).

No relevant multicollinearity was detected, with all variance inflation factors between 1.03 and 1.17.

Thus, the number of recovery events was sufficient relative to the number of estimated parameters, and neither the global nor PAIS-specific tests indicated a clear violation of the proportional-hazards assumption. The adjusted Cox model was therefore retained without the inclusion of time-varying coefficients.

## Overall assessment

The diagnostic and sensitivity analyses identified some deviations from the assumptions of the simplest regression specifications. These concerned primarily non-proportional effects of selected adjustment variables in the ordinal model and moderate heteroscedasticity and non-normality in the linear model. Importantly, the assumptions were supported for the PAIS-group effects that formed the focus of interpretation, and more flexible sensitivity models yielded virtually identical substantive conclusions.

The partial proportional-odds model reproduced the PAIS-group odds ratios from the main proportional-odds model, while the zero-truncated negative binomial model reproduced the ordering and strength of the PAIS-group associations observed in the linear model. The proportional-hazards assumption was not clearly violated in the Cox model. Taken together, these analyses support retaining the prespecified main models for their interpretability.

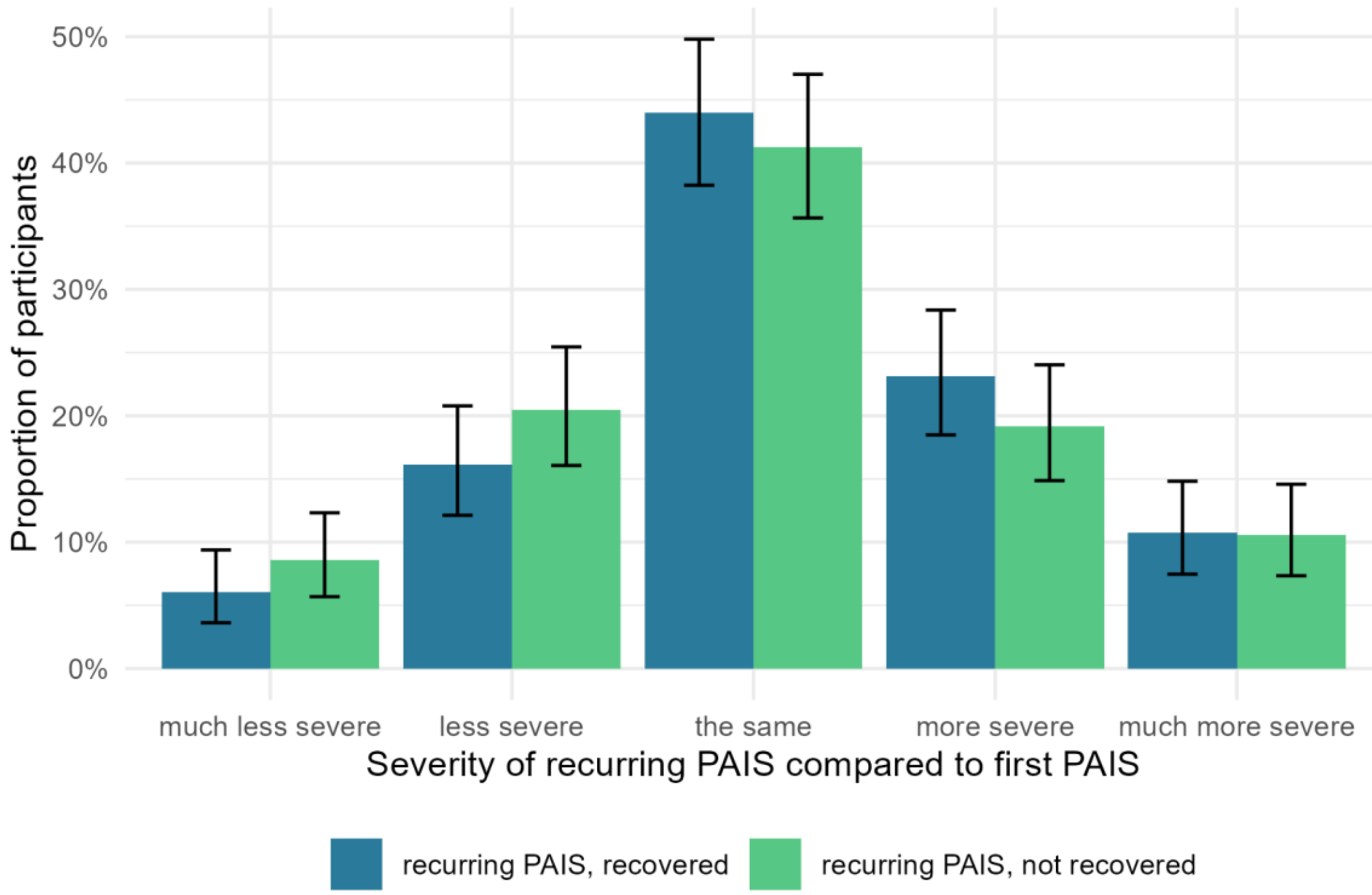


*Figure S3: Self-assessed severity of recurring PAIS compared to first PAIS episode among those reporting a recurring PAIS episode, compared for recovered vs. non-recovered before the new infection (proportion in % [95%-CI], N = 601 of 611 participants who received version 2 of the follow-up and reported a repeated PAIS episode. 10 participants were excluded due to a missing answer.).*

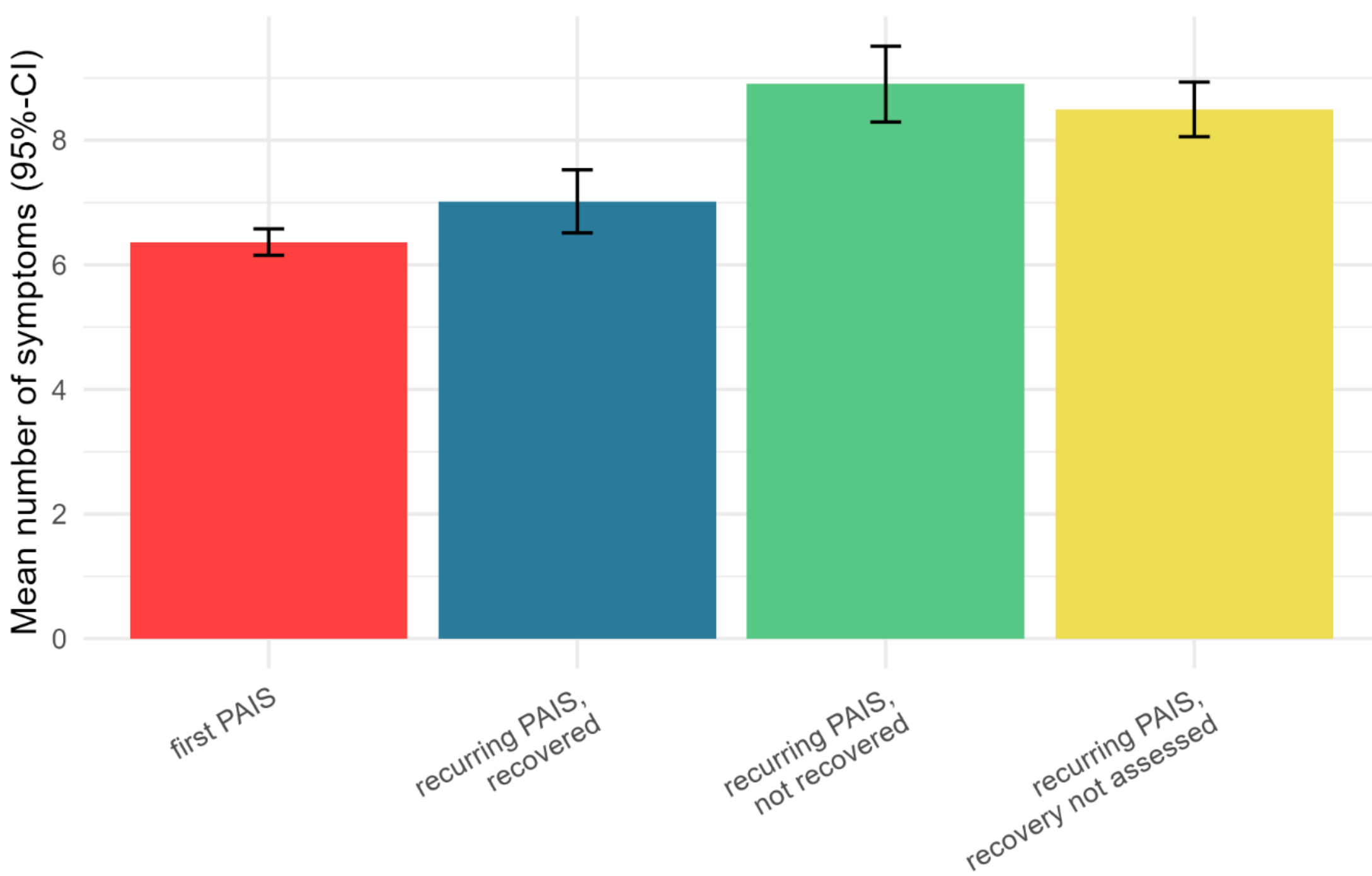


*Figure S4: Mean [95%-CI] number of symptoms compared between first and recurring PAIS episodes (N = 2,801).*

*Table S3: Factor loadings ≥ 0.35 from exploratory factor analysis with 6 factors, maximum likelihood method and oblimin rotation for all 24 symptom variables.*

| **Symptom** | **Fatigue & Exertional Intolerance** | **Cardio-vascular** | **Gastro-intestinal** | **Cognitive** | **Neuro-muscular** | **Pain** |
|---|---|---|---|---|---|---|
| Abdominal pain | – | – | 0.37 | – | – | – |
| Back pain | – | – | – | – | – | 0.50 |
| Pain in the arms, legs, or joints | – | – | – | – | – | 0.61 |
| Menstrual pain or PMS | – | – | – | – | – | – |
| Pain or problems during sexual intercourse | – | – | – | – | – | – |
| Headache | – | – | – | – | – | 0.38 |
| Chest pain | – | – | – | – | – | – |
| Vertigo | – | 0.46 | – | – | – | – |
| Fainting spell | – | – | – | – | – | – |
| Heart palpitations or rapid heartbeat | – | 0.54 | – | – | – | – |
| Shortness of breath | 0.42 | – | – | – | – | – |
| Constipation, IBS, or diarrhoea | – | – | 0.75 | – | – | – |
| Nausea, flatulence, or digestive problems | – | – | 0.85 | – | – | – |
| Sleep disturbances | 0.42 | – | – | – | – | – |
| Fatigue or lack of energy | 0.85 | – | – | – | – | – |
| Rapid exhaustion | 0.90 | – | – | – | – | – |
| Concentration difficulties | – | – | – | 0.73 | – | – |
| Memory impairment | – | – | – | 0.68 | – | – |
| Reduced physical performance | 0.70 | – | – | – | – | – |
| Hypersensitivity to noise or light | – | – | – | – | – | – |
| Circulatory problems | – | 0.66 | – | – | – | – |
| Tingling, burning, or numbness | – | – | – | – | 0.61 | – |
| Muscle twitching | – | – | – | – | 0.65 | – |
| Muscle weakness | – | – | – | – | 0.41 | – |
